# Resistance Exercise and Creatine Supplementation on Fat Mass in Adults < 50 Years of Age: A Systematic Review and Meta-Analysis

**DOI:** 10.3390/nu15204343

**Published:** 2023-10-12

**Authors:** Darren G. Candow, Konstantinos Prokopidis, Scott C. Forbes, Flavia Rusterholz, Bill I. Campbell, Sergej M. Ostojic

**Affiliations:** 1 Faculty of Kinesiology and Health Studies, University of Regina, Regina, SK S4S 0A2, Canada; frw167@uregina.ca; 2 Department of Musculoskeletal and Ageing Science, University of Liverpool, Liverpool L69 3BX, UK; k.prokopidis@liverpool.ac.uk; 3 Department of Physical Education, Faculty of Education, Brandon University, Brandon, MB R7A 6A9, Canada; forbess@brandonu.ca; 4 College of Education, University of South Florida, Tampa, FL 33620, USA; 5 Department of Nutrition and Public Health, University of Agder, 4630 Kristiansand, Norway; sergej.ostojic@chess.edu.rs

**Keywords:** body composition, ergogenic aids, adipose tissue, strength training

## Abstract

The combination of resistance exercise and creatine supplementation has been shown to decrease body fat percentage in adults ≥ 50 years of age. However, the effect on adults < 50 years of age is currently unknown. To address this limitation, we systematically reviewed the literature and performed several meta-analyses comparing studies that included resistance exercise and creatine supplementation to resistance exercise and placebo on fat mass and body fat percentage Twelve studies were included, involving 266 participants. Adults (<50 years of age) who supplemented with creatine and performed resistance exercise experienced a very small, yet significant reduction in body fat percentage (−1.19%, *p* = 0.006); however, no difference was found in absolute fat mass (−0.18 kg, *p* = 0.76). Collectively, in adults < 50 years of age, the combination of resistance exercise and creatine supplementation produces a very small reduction in body fat percentage without a corresponding decrease in absolute fat mass.

## 1. Introduction

There has been a significant increase in the prevalence of adiposity in young adults [1] which could lead to the development of adverse health conditions such as obesity, cardiovascular disease, and type 2 diabetes later in life [2,3]. From an overall health and longevity perspective, lifestyle interventions that help regulate fat mass are likely important for promoting a healthier metabolic phenotype over time [4,5]. 

A recent systematic review and meta-analysis involving over 800 healthy adults (≥19 years) showed that resistance exercise (≥4 times per week for up to 2 years) decreased fat mass by 0.55 kg (95% CI: −0.75 to −0.34; *p* < 0.0001) and body fat percentage by 1.46% (95% CI: −1.78 to −1.14; *p* < 0.0001) over time [6]. These small, yet beneficial changes, may be related to the stimulating effects of resistance exercise on the resting metabolic rate [7], excess post-oxygen consumption [8], and circulating levels of non-esterified fatty acids and by decreasing the respiratory quotient (indicating increased adipocyte lipolysis and/or intramuscular triglyceride oxidation) [9]. In addition to these small benefits of resistance exercise, there is some evidence that creatine supplementation may also lead to a reduction in body fat over time. We previously performed a meta-analysis showing that healthy older adults (n = 609; 19 studies; ≥50 years) who supplemented with creatine (≥2 g/day) and performed resistance exercise (2–3 times/week for up to 1 year) experienced a significant reduction in body fat percentage (0.55%; CI: −1.08 to −0.03; *p* = 0.04), but no differences were observed in regard to fat mass (−0.50 kg; 95% CI: −1.15 to 0.15; *p* = 0.13) compared to resistance exercise alone [10]. However, the generalizability of these findings is limited because older adults have a high degree of variability in their responsiveness and adherence to resistance exercise and creatine supplementation [11]. 

Interestingly, in children (n = 9) suffering from cancer (acute lymphoblastic leukemia), creatine significantly reduced body fat percentage over time (*p* < 0.05) [12], whereas other studies revealed no effect [13,14,15,16,17,18,19,20,21,22,23,24]. A limitation of most individual studies is that it is typically difficult to obtain adequate statistical power to detect the small differences between creatine and placebo over time due to small sample sizes. Combining studies into a meta-analysis helps overcome this limitation by assessing a large cohort of individuals. However, the meta-analytic effects of resistance exercise and creatine supplementation in adults < 50 years of age are currently unknown. This is important to determine because a common belief held by many exercising individuals is that creatine supplementation may increase fat mass over time [25], which is likely a deterrent to supplement with creatine. Therefore, the purpose of this systematic review and meta-analysis was to determine the effects of resistance exercise and creatine supplementation vs. resistance exercise alone on measures of fat mass (i.e., absolute body fat mass and body fat percentage) in adults < 50 years of age, while accounting for several confounders, including creatine dose and duration, and health status.

## 2. Materials and Methods

The PRISMA (preferred reporting items for systematic reviews and meta-analyses) standards were followed to conduct this systematic review and meta-analysis [26] and the protocol was registered in the PROSPERO (International Prospective Register of Systematic Reviews) database (CRD:42023416700).

### 2.1. Search Strategy

From the inception to April 2023, two separate reviewers (K.P. and F.R.) searched PubMed, Scopus, Web of Science, and the Cochrane library, using the following keywords: “creatine supplementation” OR “creatine” OR “creatine monohydrate” AND “body fat*” OR “body composition”. The following inclusion criteria were used: (1) studies had to be randomized controlled trials (RCTs); (2) the mean age of participants was <50 years irrespective of health status; (3) the intervention group was receiving creatine monohydrate and resistance exercise, and the comparator group was receiving resistance exercise with placebo; (4) the evaluation of fat mass was performed via dual X-ray absorptiometry (DXA), bioelectrical impedance (BIA), hydrodensitometry, magnetic resonance imaging (MRI), a computed tomography (CT) scan, or air displacement plethysmography (Bod Pod); (5) there was a minimum study duration of 4 weeks; and all of these were (6) irrespective of the language written. Studies were excluded if they: (1) were not RCTs; (2) provided only the abstract; (3) had subjects with any kind of dietary restrictions (i.e., vegans/vegetarians); or (4) were articles written by the same authors using identical populations that may have included data related to our outcomes of interest.

### 2.2. Data Extraction and Risk of Bias

Data was independently extracted by two investigators (K.P. and F.R.). The name of the first author, publication date, country of origin, study design, participant age, sex, and health status, sample size, outcomes assessed, dose and duration of creatine supplementation, fat mass assessment tool, and dietary intake assessment were among the information that was extracted. A third investigator (D.G.C.) settled disagreements between the authors. Version 2 of the Cochrane risk-of-bias 2 instrument for randomized trials (RoB2) was used to evaluate the quality of the included studies, and it was reviewed by two independent reviewers (K.P. and S.C.F.). The appraisal of the risk of bias using the RoB2 tool included the assessment of the following domains of bias in RCTs: (1) the randomization process, (2) the deviations from intended interventions, (3) the missing outcome data, (4) the measurement of the outcome, and (5) the selection of the reported result. The study quality was categorized as either having a low risk of bias, considerable concerns, or a high risk of bias using the RoB2 tool rating system.

### 2.3. Statistical Analysis

The mean differences between groups were calculated by comparing changes in outcomes from baseline to follow-up, treating quantitative data as continuous measurements. Standardized mean differences were employed when measurement units were inconsistent (e.g., body fat percentage changes mixed with absolute body fat kilogram changes) and could not be changed to the units needed for the analyses. The inverse-variance approach and the random-effects model were used to determine statistical significance. Standard deviations and missing data for any changes between the baseline and follow-up outcome data were determined by deriving a correlation coefficient of 0.5, considering that a value of standard deviation change from baseline derived from an included study was not provided.

Utilizing the overlap of their 95% confidence intervals (CIs) and expressing the results as a measurement of Cochran’s Q (χ^2^ test) and I^2^, the statistical heterogeneity of the outcome measurements across the included studies was evaluated. Low heterogeneity was considered when the I^2^ levels were <50%, moderate heterogeneity between 50% and 74.9%, and high heterogeneity ≥ 75%. Subgroup analyses based on age (<40 years vs. 41–49 years), sex (males only vs. females only vs. mixed sexes), fat mass assessment tool (DXA vs. BIA vs. Hydrodensitometry vs. Bod Pod), body mass index (BMI) (<25 kg/m^2^ vs. ≥25 kg/m^2^), creatine monohydrate duration (<8 weeks vs. ≥8 weeks) and dose (≤5 g/d vs. >5 g/d), and resistance exercise frequency (≤3x/week vs. >3x/week) were performed. Additionally, sensitivity analyses were performed to evaluate the robustness of the reported statistical results by discounting the effects of a lack of dietary intake assessment, participants with comorbidities, and studies with an increased risk of bias. The meta-analyses were synthesized using Cochrane’s Review Manager (RevMan 5.4.1) software.

## 3. Results

### 3.1. Literature Search

**Figure 1:**
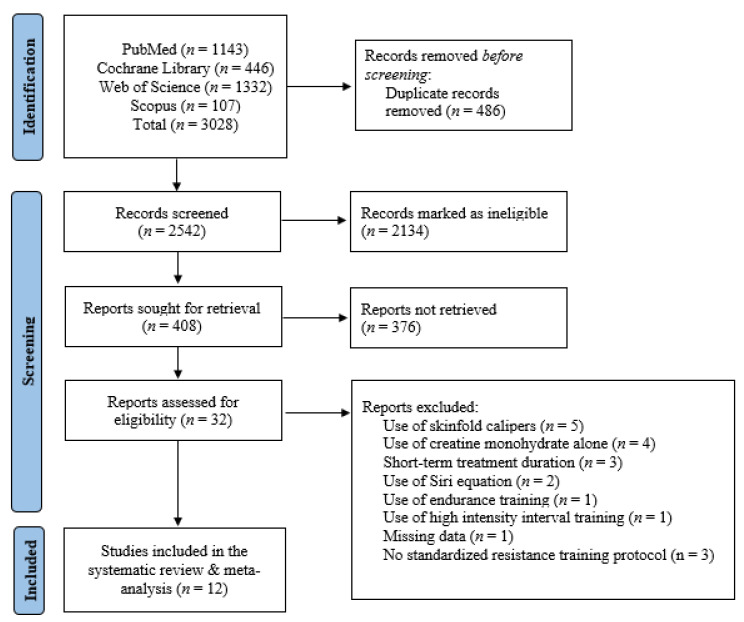
Study selection flow chart.

In the initial literature search, 3028 publications were found. Of these, 486 duplicate publications were eliminated, leaving 2542 distinct publications, from which 2134 were deemed ineligible based on titles and abstracts, and another 376 publications were not retrieved due to irrelevant study designs and outcomes of interest. In total, 32 RCTs investigating the effects of creatine monohydrate on body fat in adults aged < 50 years were found. After further examination of the remaining publications, five of these used skinfold calipers for the measurement of body fat, four used creatine monohydrate in the absence of resistance training, three had a short-term treatment duration (<4 weeks), two used the Siri equation to quantify body fat, one had inadequate data, one used endurance training, one used high-intensity interval training, and three did not have a standardized resistance training protocol. Overall, 12 RCTs were included in this systematic review and meta-analysis (Figure 1), involving 266 participants (130 in the creatine monohydrate and resistance exercise group and 133 in the placebo and resistance exercise group). A detailed description of the included studies is depicted in Table 1.

### 3.2. Creatine Supplementation and Body Fat Changes

**Figure 2:**
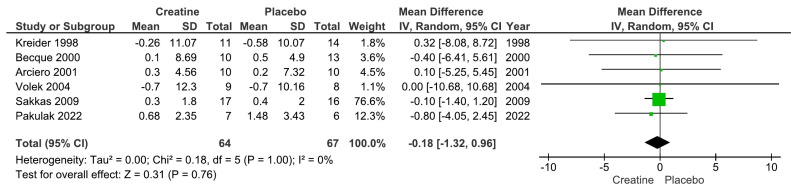
Forest plots for changes in absolute fat mass (kg) [13,14,18,19,21,22].

**Figure 3:**
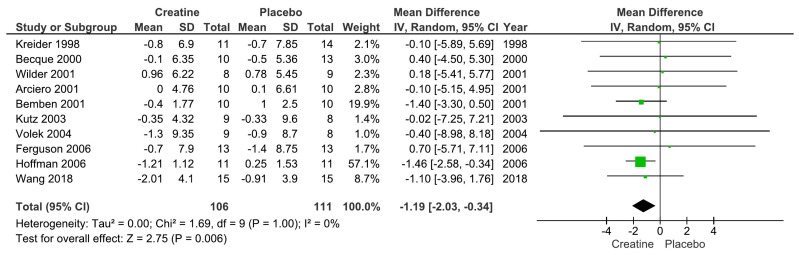
Forest plot for changes in body fat percentage [13,14,16,17,18,20,22,23,24,27].

Our main analysis showed that creatine supplementation did not significantly impact changes in absolute fat mass (kg) over time (k = 6; MD = −0.18; 95%CI, −1.32 to 0.96; I^2^ = 0%; *p* = 0.76) (Figure 2). However, creatine did produce a significant reduction in body fat percentage over time (k = 10; MD = −1.19; 95% CI, −2.03 to −0.34; I^2^ = 0%; *p* = 0.006) (Figure 3). 

The subgroup analysis based on age (<40 years vs. 41–49 years) showed that creatine supplementation did not influence fat mass more than placebo (<40 years: SMD = −0.18; 95% CI, −0.44 to 0.08; I^2^ = 0%; *p* = 0.18 vs. 41–49 years: SMD = −0.05; 95% CI, −0.73 to 0.63; *p* = 0.88) (Figure S1). Similar results were found with regards to the fat mass assessment tool (DXA: SMD = −0.13; 95% CI, −0.46 to 0.20; I^2^ = 0%; *p* = 0.44 vs. BIA: SMD = −0.27; 95% CI, −0.99 to 0.45; *p* = 0.47 vs. hydrodensitometry: SMD = −0.17; 95% CI, −0.62 to 0.29; I^2^ = 0%; *p* = 0.47 vs. Bod Pod: SMD = −0.26; 95% CI, −1.35 to 0.84; *p* = 0.65) (Figure S2), BMI (<25 kg/m^2^: SMD = −0.14; 95% CI, −0.45 to 0.16; I^2^ = 0%; *p* = 0.36 vs. ≥25 kg/m^2^: SMD = −0.20; 95% CI, −0.61 to 0.22; I^2^ = 6%; *p* = 0.35) (Figure S3), sex (Females only: SMD = 0.08; 95% CI, −0.69 to 0.85; *p* = 0.84 vs. Males only: SMD = −0.19; 95% CI, −0.45 to 0.08; I^2^ = 0%; *p* = 0.17 vs. Mixed: SMD = −0.26; 95% CI, −1.35 to 0.84; *p* = 0.65) (Figure S4), creatine dose (<5 g: SMD = −0.12; 95% CI, −0.43 to 0.18; I^2^ = 0%; *p* = 0.43 vs. ≥5 g: SMD = −0.24; 95% CI, −0.65 to 0.17; I^2^ = 2%; *p* = 0.26) (Figure S5) and duration of supplementation (<8 weeks: SMD = −0.08; 95% CI, −0.41 to 0.25; I^2^ = 0%; *p* = 0.63 vs. ≥8 weeks: SMD = −0.28; 95% CI, −0.69 to 0.13; I^2^ = 20%; *p* = 0.18) (Figure S6), and resistance exercise frequency (≤3x/week: SMD = −0.07; 95% CI, −0.41 to 0.26; I^2^ = 0%; *p* = 0.67 vs. >3x/week: SMD = −0.27; 95% CI, −0.63 to 0.09; I^2^= 2%; *p* = 0.14) (Figure S7).

The sensitivity analysis excluding participants with health conditions (Healthy participants: SMD = −0.18; 95% CI, −0.44 to 0.08; I^2^ = 0%; *p* = 0.18 vs. Unhealthy participants: SMD = −0.05; 95% CI, −0.73 to 0.63; *p* = 0.88) (Figure S8), studies that did not assess for dietary intake (Assessment: SMD = −0.20; 95% CI, −0.52 to 0.11; I^2^ = 0%; *p* = 0.20 vs. No assessment: SMD = −0.10; 95% CI, −0.49 to 0.29; I^2^ = 0%; *p* = 0.61) (Figure S9), and studies with increased risk of bias (SMD = −0.19; 95% CI, −0.45 to 0.08; I^2^ = 0%; *p* = 0.17) (Figure S10) did not alter any of the findings. 

### 3.3. Risk of Bias Assessment

**Figure 4:**
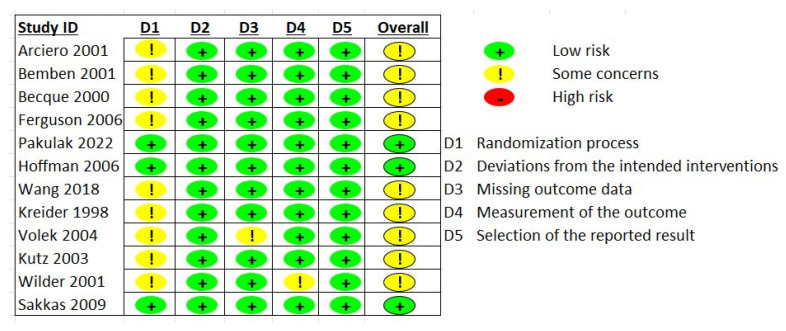
Risk of bias assessment [13,14,16,17,18,19,20,21,22,23,24,27].

Four of the studies were classified as having a low risk of bias [16,17,19,21], six studies had a moderate risk [13,18,22,23,24,27], and two studies had a high risk of bias [14,20]. These concerns primarily arose due to the absence of specific details regarding randomization procedures or treatment allocation, considering that two studies did not report whether the participants were randomized [14,20]. Lastly, in one study, the supplement was provided in a single-blind fashion [24]. A detailed description of the risk of bias assessment is depicted in Figure 4.

## 4. Discussion

This is the first meta-analysis to examine the efficacy of resistance exercise and creatine supplementation vs. resistance exercise alone on measures of fat mass in adults < 50 years of age. Results showed that the combination of resistance exercise and creatine supplementation (≥4 weeks) significantly reduced body fat percentage by 1.19%, without a corresponding reduction in absolute fat mass (−0.18 kg), compared to resistance exercise alone. Variables such as age (<40 years; 41–49 years), sex (females only; males only; females and males combined), fat mass assessment tool, creatine dosage (<5 g; ≥5 g) and duration of creatine supplementation (<8 weeks; ≥8 weeks), and resistance training frequency (≤3x/week; >3x/week) did not alter these findings. 

The minimal reduction in body fat percentage from resistance exercise and creatine supplementation in adults < 50 years is comparable to our previous meta-analysis findings in adults ≥ 50 years (body fat percentage: −0.55) [10]. Collectively, these findings help refute the common belief held by many exercising individuals that creatine supplementation increases fat mass over time [25]. However, these results are likely clinically and practically insignificant, considering that the very small reduction in body fat percentage did not correspond to a significant reduction in absolute fat mass. Although speculative, the very small reduction in body fat percentage from creatine could be attributable to changes in lean mass and/or muscle accretion over time. For example, several meta-analyses have been performed collectively showing that the combination of creatine supplementation and resistance exercise increases measures of whole-body lean tissue mass by ~1.37 kg (as measured by dual-energy X-ray absorptiometry, air-displacement plethysmography, hydrodensitometry, and bioelectrical impedance analysis) compared to placebo and resistance exercise [28,29,30,31,32]. Furthermore, Burke et al. [33] performed a systematic review and meta-analysis involving 10 studies and found significant improvements in direct measures of limb muscle hypertrophy (0.10–0.16 cm; as measured using ultrasound and peripheral quantitative computed tomography {pQCT}) in the upper- and lower-body from creatine supplementation and resistance exercise compared to resistance exercise and placebo. Interestingly, the lone study that used pQCT showed that creatine supplementation (52 weeks) increased lower-limb muscle density (Δ + 0.83 ± 1.15 mg·cm^−3^; *p* = 0.016) compared to placebo (Δ −0.16 ± 1.56 mg·cm^−3^). Mechanistically, these lean tissue and regional muscle improvements may be related to creatine increasing cellular hydration status, high-energy phosphate metabolism (phosphocreatine content and recovery), glycogen synthesis, satellite cell proliferation and activity, growth factor production and expression (i.e., insulin-like growth factor-1), myogenic transcription factor expression (Myf5, Mrf4, MyoD, and myogenin), protein kinases downstream in the mammalian target of the rapamycin (mTOR) signaling pathway which are involved in translation and decreasing measures of inflammation, oxidative stress (reactive oxygen species), and protein catabolism (whole-body leucine oxidation and urinary excretion of 3-methylhistidine) [31]. The significant increases in whole-body lean tissue mass, limb muscle hypertrophy, and muscle density from creatine supplementation may increase energy expenditure which could reduce body fat percentage over time [34]. However, even with the inclusion of increased weekly resistance exercise sessions, we did not observe an additional response in body fat levels. Our results indirectly support this notion as there was a statistically significant reduction in body fat percentage with only a small, non-significant change in fat mass over time. Unfortunately, the mechanistic effects of creatine, with and without resistance exercise, in healthy adults (≥18 years) are unknown. 

### Limitations

Results from this meta-analysis are likely affected by the measurement errors of the body composition assessment tool used and subsequent changes in lean tissue and/or total body water. Further, multiple studies did not control the dietary intake of creatine which may have influenced our findings. Moreover, considering the fundamental physiological differences underpinning men and women, we could not establish a subgroup analysis or a meta-regression to account for this confounder. Another limitation can also be attributed to the varied training status of the participants and the variability pertaining to the intensity of exercise protocols. Finally, no mechanisms of lipolysis or beta-oxidation were determined in this meta-analysis. 

## 5. Conclusions

In adults < 50 years of age, the combination of resistance exercise and creatine supplementation results in a minimal reduction in body fat percentage (1.19%) with no effect on absolute fat mass compared to resistance exercise alone. Creatine supplementation combined with resistance exercise does not increase fat mass in adults < 50 years of age. 

## Figures and Tables

**Table 1:** Summary of study characteristics.

First Author, Year	Population	Supplement Dose	Fat Mass Assessment Tool	Duration	RT Protocol	Baseline Measures	Follow-Up Results
Arciero et al. 2001 [13]	N = 30; healthy males (21 ± 3 year)	CR: 20 g/day (5 g 4 × daily) for 5 days and then 10 g/day (5 g 2 × daily) for the remainder; PLA: same dosing as CR (dextrose)	DEXA	28 days	RT 3x/week; 2 sets of 10 at 70% 1RM and 1 set performed to failure.	Intervention %BF: 16.4 ± 4.9 Fat Mass (kg): 12.8 ± 4.7 FFM (kg): 63.0 ± 2.8 Control %BF: 16.3 ± 6.4 Fat Mass (kg): 12.9 ± 7.0 FFM (kg): 62.3 ± 6.5	Intervention %BF: 16.4 ± 4.6 Fat Mass (kg): 13.1 ± 4.4 FFM (kg): 64.7 ± 3.6 Control %BF: 16.4 ± 6.8 Fat Mass (kg): 13.1 ± 7.6 FFM (kg): 62.5 ± 6.5
Becque et al. 2000 [14]	N = 23; healthy males with at least 1 y weight training experience (CR: n = 10; PLA: n = 13); age: 21.5 ± 2.7 year	CR: 20 g/day (5 g 4 × daily) for 5 days and then 2 g/day for the remainder; PLA: same dosing as CR (sucrose)	Hydrodensitometry	6 weeks	RT 2x/week (arm flexor: preacher curl)	Intervention %BF: 17.8 ± 6.9 Fat Mass (kg): 15.6 ± 9.0 FFM (kg): 71.2 ± 10 Control %BF: 16 ± 5.5 Fat Mass (kg): 13.1 ± 5.0 FFM (kg): 68.6 ± 5.8	Intervention %BF: 17.7 ± 5.6 Fat Mass (kg): 15.7 ± 6.7 FFM (kg): 72.8 ± 10.1 Control %BF: 16.5 ± 5.2 Fat Mass (kg): 13.6 ± 4.8 FFM (kg): 68.5 ± 5.9
Bemben et al. 2001 [27]	N = 25 NCAA Division 1 football athletes; (PLA: n = 8; CR: n = 9; control: n = 8); age: 18–22 year	CR: 20 g/day (4 equal doses) for 5 d followed by 5 g/day; PLA: same dose (sodium phosphate)	Hydrodensitometry	9 weeks	RT 4x/week split routine	Intervention %BF: 12.6 ± 1.9 LBM (kg): 77.1 ± 4.2 Control %BF: 13.9 ± 2.5 LBM (kg): 78.2 ± 2.2	Intervention %BF: 12.2 ± 1.6 LBM (kg): 80.0 ± 5.0 Control %BF: 14.9 ± 2.5 LBM (kg): 78.0 ± 2.2
Ferguson and Syrotuik 2006 [16]	N = 26 healthy recreational strength-trained women; age: 18–35 year	CR: 0.3 g/kg/day for 7 days and then 0.03 g/kg/day for the remainder or PLA	DEXA	10 weeks	RT 4x/week split routine	Intervention %BF: 30.9 ± 8.0 LBM (kg): 42.8 ± 4.153 Control %BF: 29.5 ± 8.6 LBM (kg): 43.83 ± 4.153	Intervention %BF: 30.2 ± 7.8 LBM (kg): 43.88 ± 4.003 Control %BF: 28.1 ± 8.9 LBM (kg): 44.75 ± 4.695
Hoffman et al. 2006 [17]	N = 33 male strength power athletes; age: not reported	CR: 10.5 g/day or PLA: Dextrose	DEXA	10 weeks	RT 4x/week split routine	-	Intervention Δ%BF: −1.21 ± 1.12 ΔLBM (kg): 1.74 ± 1.72 Control Δ%BF: 0.25 ± 1.53 ΔLBM (kg): −0.44 ± 1.62
Kreider et al. 1998 [18]	N = 25 NCAA Division 1 football athletes; age: 19.9 ± 0.3 year	CR: 15.75 g/day or PLA	DEXA	28 days	RT: 4x/week + agility/sprint training 3x/week	Intervention %BF: 17.0 ± 6.8 Fat Mass (kg): 16.2 ± 10.06 Control %BF: 18.0 ± 8.0 Fat Mass (kg): 17.25 ± 10.27	Intervention %BF: 16.2 ± 7.0 Fat Mass (kg): 15.94 ± 11.86 Control %BF: 17.3 ± 7.7 Fat Mass (kg): 16.67 ± 9.86
Kutz and Gunter 2003 [20]	N = 17 active males; age: 22.9 ± 4.9 year	CR: 30 g/day for 2 weeks followed by 15 g/day for the remainder or PLA (dextrose)	Hydrodensitometry	4 weeks	RT: 2x/week lower body only	Intervention %BF: 14.32 ± 4.58 Control %BF: 15.45 ± 9.35	Intervention %BF: 13.97 ± 4.00 Control %BF: 15.12 ± 9.83
Pakulak et al. 2022 [19]	N = 28 resistance-trained males and females (CR: n = 7; PLA: n = 6); age: 18–38 year	CR: 0.1 g/kg/day or PLA (maltodextrin)	Air displacement plethysmography	6 weeks	RT: 5–6x/week split routine	Intervention Fat Mass (kg): 16.8 ± 3.4 FFM (kg): 59.1 ± 9.9 Control Fat Mass (kg): 14.3 ± 7.7 FFM (kg): 60.7 ± 13.8	Intervention ΔFat Mass (kg): 0.68 ± 0.81 ΔFFM (kg): 1.28 ± 0.69 Control ΔFat Mass (kg): 1.48 ± 1.18 ΔFFM (kg): 0.78 ± 1.23
Sakkas et al. 2009 [21]	N = 40 HIV-positive men	CR: 20 g/day for 5 days followed by 4.8 g/day for the remainder or PLA	DEXA	14 weeks	RT: 3x/week	Intervention Fat Mass (kg): 13.7 ± 6.4 LBM (kg): 56.0 ± 7.3 Control Fat Mass (kg): 11.8 ± 4.8 LBM (kg): 57.4 ± 6.6	Intervention ΔFat Mass (kg): 0.3 ± 1.8 Δ LBM (kg): 2.3 ± 1.4 Control ΔFat Mass (kg): 0.4 ± 2.0 Δ LBM (kg): 0.9 ± 1.4
Volek et al. 2003 [22]	N = 17 healthy males; age: 21 ± 3 year	CR: 0.3 g/kg/day for 7 days followed by 0.05 g/kg/day for the remainder or PLA (cellulose)	DEXA	4 weeks	RT: 5x/week	Intervention %BF: 17.4 ± 9.2 Fat Mass (kg): 16.5 ± 12.1 LBM (kg): 67.2 ± 5.6 Control %BF: 20.2 ± 8.8 Fat Mass (kg): 18.8 ± 10.4 LBM (kg): 66.9 ± 5.4	Intervention %BF: 16.1 ± 9.5 Fat Mass (kg): 15.8 ± 12.5 LBM (kg): 70.6 ± 5.8 Control %BF: 19.3 ± 8.6 Fat Mass (kg): 18.1 ± 9.9 LBM (kg): 68.9 ± 6.0
Wang et al. 2018 [23]	N = 30 male athletes (baseball, basketball, tchoukball); age: 20 ± 2 year	CR: 20 g/day for 6 days followed by 2 g/day for the remainder or PLA (cellulose)	Bioelectrical impedance analysis	4 weeks	RT: Complex training including heavy resistance training and plyometrics 3x/week	Intervention %BF: 15.78 ± 4.18 FFM (kg): 57.07 ± 4.84 Control %BF: 13.67 ± 4.37 FFM (kg): 60.47 ± 9.55	Intervention %BF: 13.77 ± 4.01 FFM (kg): 58.97 ± 5.18 Control %BF: 12.76 ± 3.13 FFM (kg): 61.58 ± 9.17
Wilder et al. 2001 [24]	N = 25 division 1A collegiate football players; age: 20 ± 2 year	CR: 20 g/day for 6 days followed by 5 g/day for the remainder or 3 g/day or PLA (cellulose)	Hydrodensitometry	4 weeks	RT: Complex training including heavy resistance training and plyometrics 3x/week	Intervention %BF: 14.85 ± 5.92 FFM (kg): 81.67 ± 10.88 Control %BF: 13.75 ± 5.85 FFM (kg): 81.53 ± 8.63	Intervention %BF: 15.81 ± 5.28 FFM (kg): 84.64 ± 9.79 Control %BF: 14.53 ± 5.08 FFM (kg): 82.59 ± 7.29

Data are expressed as the mean ± SD. %BF, body fat percentage; CR, creatine; DEXA, dual X-ray absorptiometry; FFM, fat-free mass; LBM, lean body mass; PLA, placebo; and RT, resistance training.

## Data Availability

Not applicable.

## Supplementary Materials

The following supporting information can be downloaded at: https://www.mdpi.com/article/10.3390/nu15204343/s1, Figure S1: Subgroup analysis based on age (<40 years vs. 41–49 years); Figure S2: Subgroup analysis according to fat mass assessment tool; Figure S3: Subgroup analysis based on BMI (<25 kg/m^2^ vs. ≥25 kg/m^2^); Figure S4: Subgroup analysis based on sex (females vs. males vs. mixed); Figure S5: Subgroup analysis based on creatine dose (<5 g vs. ≥5 g); Figure S6: Subgroup analysis based on duration of supplementation (<8 weeks vs. ≥8 weeks); Figure S7: Subgroup analysis based on resistance exercise frequency (≤3x/week vs. >3x/week); Figure S8: Sensitivity analysis excluding participants with health conditions; Figure S9: Sensitivity analysis excluding studies that did not assess dietary intake; Figure S10: Sensitivity analysis excluding studies with increased risk of bias.
