# Supplementary material for: Resistance Exercise and Creatine Supplementation on Fat Mass in Adults < 50 Years of Age: A Systematic Review and Meta-Analysis
Source: Nutrients. 2023 Oct 12;15(20):4343. doi: 10.3390/nu15204343 (PMC10609732; doi:10.3390/nu15204343)
Supplement: Supplementary file 1 [file nutrients-15-04343-s001.zip › nutrients-2647838-supplementary.pdf]

Study or Subgroup	Creatine Mean	Creatine SD	Creatine Total	Placebo Mean	Placebo SD	Placebo Total	Weight	Std. Mean Difference IV, Random, 95% CI	Year
**1.1.1 Below 40 years old**									
Kreider 1998	-0.26	11.07	11	-0.58	10.07	14	9.6%	0.03 [-0.76, 0.82]	1998
Becque 2000	0.1	8.69	10	0.5	4.9	13	8.8%	-0.06 [-0.88, 0.77]	2000
Wilder 2001	0.96	6.22	8	0.78	5.45	9	6.6%	0.03 [-0.92, 0.98]	2001
Arciero 2001	0.3	4.56	10	0.2	7.32	10	7.8%	0.02 [-0.86, 0.89]	2001
Bemben 2001	-0.4	1.77	10	1	2.5	10	7.3%	-0.62 [-1.52, 0.28]	2001
Kutz 2003	-0.35	4.32	9	-0.33	9.6	8	6.6%	-0.00 [-0.95, 0.95]	2003
Volek 2004	-0.7	12.3	9	-0.7	10.16	8	6.6%	0.00 [-0.95, 0.95]	2004
Ferguson 2006	-0.7	7.9	13	-1.4	8.75	13	10.1%	0.08 [-0.69, 0.85]	2006
Hoffman 2006	-1.21	1.12	11	0.25	1.53	11	7.3%	-1.05 [-1.95, -0.14]	2006
Wang 2018	-2.01	4.1	15	-0.91	3.9	15	11.5%	-0.27 [-0.99, 0.45]	2018
Pakulak 2022	0.68	2.35	7	1.48	3.43	6	5.0%	-0.26 [-1.35, 0.84]	2022
**Subtotal (95% CI)**			**113**			**117**	**87.2%**	**-0.18 [-0.44, 0.08]**	
Heterogeneity: Tau² = 0.00; Chi² = 5.98, df = 10 (P = 0.82); I² = 0%									
Test for overall effect: Z = 1.35 (P = 0.18)									
**1.1.2 40 years old and above**									
Sakkas 2009	0.3	1.8	17	0.4	2	16	12.8%	-0.05 [-0.73, 0.63]	2009
**Subtotal (95% CI)**			**17**			**16**	**12.8%**	**-0.05 [-0.73, 0.63]**	
Heterogeneity: Not applicable									
Test for overall effect: Z = 0.15 (P = 0.88)									
**Total (95% CI)**			**130**			**133**	**100.0%**	**-0.16 [-0.41, 0.08]**	
Heterogeneity: Tau² = 0.00; Chi² = 6.10, df = 11 (P = 0.87); I² = 0%									
Test for overall effect: Z = 1.31 (P = 0.19)									
Test for subgroup differences: Chi² = 0.12, df = 1 (P = 0.73), I² = 0%									

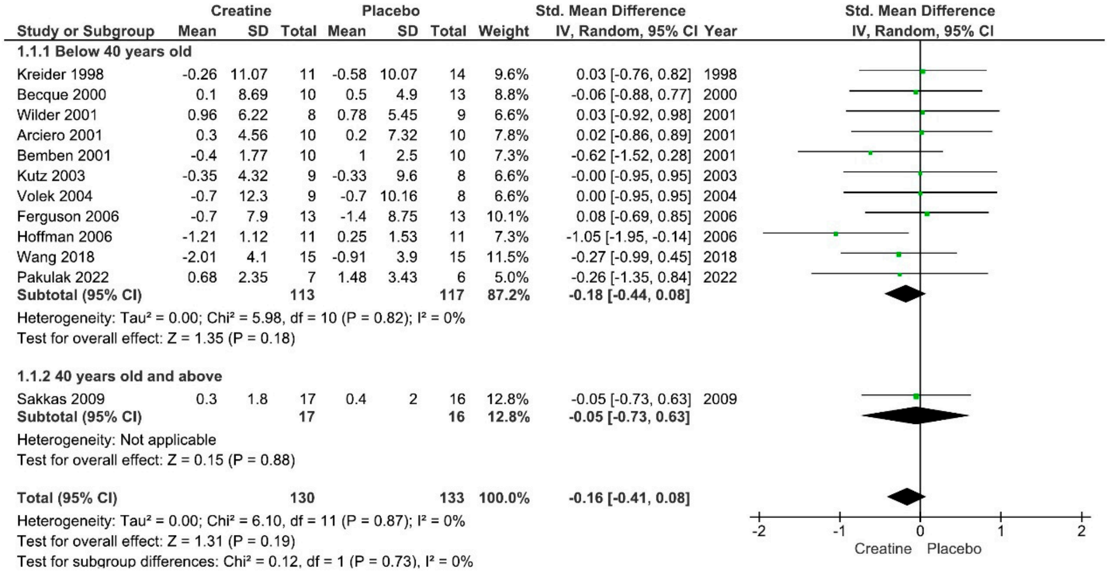

Figure S1. Subgroup analysis based on age (<40 years vs. 41-49 years).

Study or Subgroup	Creatine Mean	Creatine SD	Creatine Total	Placebo Mean	Placebo SD	Placebo Total	Weight	Std. Mean Difference IV, Random, 95% CI	Year
**1.1.1 DXA**									
Kreider 1998	-0.26	11.07	11	-0.58	10.07	14	9.6%	0.03 [-0.76, 0.82]	1998
Arciero 2001	0.3	4.56	10	0.2	7.32	10	7.8%	0.02 [-0.86, 0.89]	2001
Volek 2004	-0.7	12.3	9	-0.7	10.16	8	6.6%	0.00 [-0.95, 0.95]	2004
Ferguson 2006	-0.7	7.9	13	-1.4	8.75	13	10.1%	0.08 [-0.69, 0.85]	2006
Hoffman 2006	-1.21	1.12	11	0.25	1.53	11	7.3%	-1.05 [-1.95, -0.14]	2006
Sakkas 2009	0.3	1.8	17	0.4	2	16	12.8%	-0.05 [-0.73, 0.63]	2009
**Subtotal (95% CI)**			**71**			**72**	**54.2%**	**-0.13 [-0.46, 0.20]**	
Heterogeneity: Tau² = 0.00; Chi² = 4.64, df = 5 (P = 0.46); I² = 0%									
Test for overall effect: Z = 0.77 (P = 0.44)									
**1.1.2 BIA**									
Wang 2018	-2.01	4.1	15	-0.91	3.9	15	11.5%	-0.27 [-0.99, 0.45]	2018
**Subtotal (95% CI)**			**15**			**15**	**11.5%**	**-0.27 [-0.99, 0.45]**	
Heterogeneity: Not applicable									
Test for overall effect: Z = 0.73 (P = 0.47)									
**1.1.3 Hydrodensitometry**									
Becque 2000	0.1	8.69	10	0.5	4.9	13	8.8%	-0.06 [-0.88, 0.77]	2000
Bemben 2001	-0.4	1.77	10	1	2.5	10	7.3%	-0.62 [-1.52, 0.28]	2001
Wilder 2001	0.96	6.22	8	0.78	5.45	9	6.6%	0.03 [-0.92, 0.98]	2001
Kutz 2003	-0.35	4.32	9	-0.33	9.6	8	6.6%	-0.00 [-0.95, 0.95]	2003
**Subtotal (95% CI)**			**37**			**40**	**29.3%**	**-0.17 [-0.62, 0.29]**	
Heterogeneity: Tau² = 0.00; Chi² = 1.31, df = 3 (P = 0.73); I² = 0%									
Test for overall effect: Z = 0.72 (P = 0.47)									
**1.1.4 Bod Pod**									
Pakulak 2022	0.68	2.35	7	1.48	3.43	6	5.0%	-0.26 [-1.35, 0.84]	2022
**Subtotal (95% CI)**			**7**			**6**	**5.0%**	**-0.26 [-1.35, 0.84]**	
Heterogeneity: Not applicable									
Test for overall effect: Z = 0.46 (P = 0.65)									
**Total (95% CI)**			**130**			**133**	**100.0%**	**-0.16 [-0.41, 0.08]**	
Heterogeneity: Tau² = 0.00; Chi² = 6.10, df = 11 (P = 0.87); I² = 0%									
Test for overall effect: Z = 1.31 (P = 0.19)									
Test for subgroup differences: Chi² = 0.14, df = 3 (P = 0.99), I² = 0%									

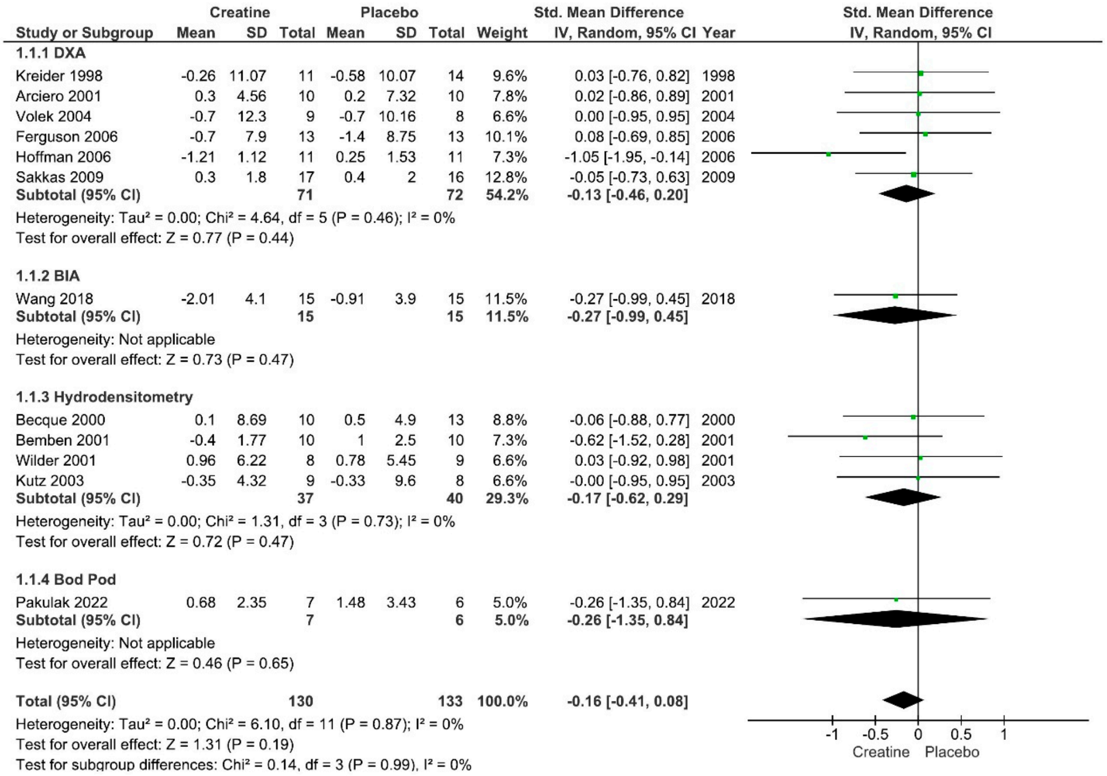

Figure S2. Subgroup analysis according to fat mass assessment tool.

Study or Subgroup	Creatine Mean	Creatine SD	Creatine Total	Placebo Mean	Placebo SD	Placebo Total	Weight	Std. Mean Difference IV, Random, 95% CI	Year
**1.1.1 BMI below 25**									
Becque 2000	0.1	8.69	10	0.5	4.9	13	8.8%	-0.06 [-0.88, 0.77]	2000
Arciero 2001	0.3	4.56	10	0.2	7.32	10	7.8%	0.02 [-0.86, 0.89]	2001
Bemben 2001	-0.4	1.77	10	1	2.5	10	7.3%	-0.62 [-1.52, 0.28]	2001
Ferguson 2006	-0.7	7.9	13	-1.4	8.75	13	10.1%	0.08 [-0.69, 0.85]	2006
Sakkas 2009	0.3	1.8	17	0.4	2	16	12.8%	-0.05 [-0.73, 0.63]	2009
Wang 2018	-2.01	4.1	15	-0.91	3.9	15	11.5%	-0.27 [-0.99, 0.45]	2018
Pakulak 2022	0.68	2.35	7	1.48	3.43	6	5.0%	-0.26 [-1.35, 0.84]	2022
**Subtotal (95% CI)**			**82**			**83**	**63.3%**	**-0.14 [-0.45, 0.16]**	
Heterogeneity: Tau² = 0.00; Chi² = 1.79, df = 6 (P = 0.94); I² = 0%									
Test for overall effect: Z = 0.92 (P = 0.36)									
**1.1.2 BMI 25 and above**									
Kreider 1998	-0.26	11.07	11	-0.58	10.07	14	9.6%	0.03 [-0.76, 0.82]	1998
Wilder 2001	0.96	6.22	8	0.78	5.45	9	6.6%	0.03 [-0.92, 0.98]	2001
Kutz 2003	-0.35	4.32	9	-0.33	9.6	8	6.6%	-0.00 [-0.95, 0.95]	2003
Volek 2004	-0.7	12.3	9	-0.7	10.16	8	6.6%	0.00 [-0.95, 0.95]	2004
Hoffman 2006	-1.21	1.12	11	0.25	1.53	11	7.3%	-1.05 [-1.95, -0.14]	2006
**Subtotal (95% CI)**			**48**			**50**	**36.7%**	**-0.20 [-0.61, 0.22]**	
Heterogeneity: Tau² = 0.01; Chi² = 4.27, df = 4 (P = 0.37); I² = 6%									
Test for overall effect: Z = 0.93 (P = 0.35)									
**Total (95% CI)**			**130**			**133**	**100.0%**	**-0.16 [-0.41, 0.08]**	
Heterogeneity: Tau² = 0.00; Chi² = 6.10, df = 11 (P = 0.87); I² = 0%									
Test for overall effect: Z = 1.31 (P = 0.19)									
Test for subgroup differences: Chi² = 0.04, df = 1 (P = 0.84), I² = 0%									

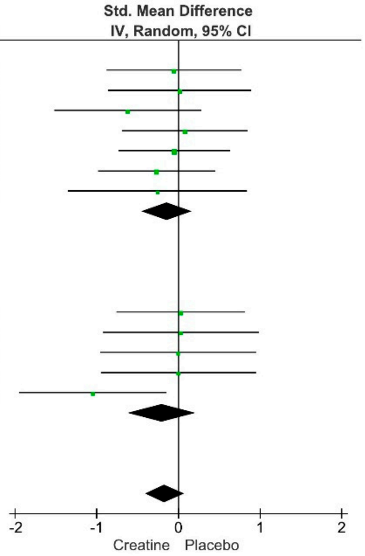

Figure S3. Subgroup analysis based on BMI (<25 kg/m^2 vs. ≥25 kg/m^2).

Study or Subgroup	Creatine Mean	Creatine SD	Creatine Total	Placebo Mean	Placebo SD	Placebo Total	Weight	Std. Mean Difference IV, Random, 95% CI	Year
**1.1.1 Females**									
Ferguson 2006	-0.7	7.9	13	-1.4	8.75	13	10.1%	0.08 [-0.69, 0.85]	2006
**Subtotal (95% CI)**			**13**			**13**	**10.1%**	**0.08 [-0.69, 0.85]**	
Heterogeneity: Not applicable									
Test for overall effect: Z = 0.21 (P = 0.84)									
**1.1.2 Males**									
Kreider 1998	-0.26	11.07	11	-0.58	10.07	14	9.6%	0.03 [-0.76, 0.82]	1998
Becque 2000	0.1	8.69	10	0.5	4.9	13	8.8%	-0.06 [-0.88, 0.77]	2000
Arciero 2001	0.3	4.56	10	0.2	7.32	10	7.8%	0.02 [-0.86, 0.89]	2001
Bemben 2001	-0.4	1.77	10	1	2.5	10	7.3%	-0.62 [-1.52, 0.28]	2001
Wilder 2001	0.96	6.22	8	0.78	5.45	9	6.6%	0.03 [-0.92, 0.98]	2001
Kutz 2003	-0.35	4.32	9	-0.33	9.6	8	6.6%	-0.00 [-0.95, 0.95]	2003
Volek 2004	-0.7	12.3	9	-0.7	10.16	8	6.6%	0.00 [-0.95, 0.95]	2004
Hoffman 2006	-1.21	1.12	11	0.25	1.53	11	7.3%	-1.05 [-1.95, -0.14]	2006
Sakkas 2009	0.3	1.8	17	0.4	2	16	12.8%	-0.05 [-0.73, 0.63]	2009
Wang 2018	-2.01	4.1	15	-0.91	3.9	15	11.5%	-0.27 [-0.99, 0.45]	2018
**Subtotal (95% CI)**			**110**			**114**	**84.9%**	**-0.19 [-0.45, 0.08]**	
Heterogeneity: Tau² = 0.00; Chi² = 5.65, df = 9 (P = 0.77); I² = 0%									
Test for overall effect: Z = 1.38 (P = 0.17)									
**1.1.3 Mixed**									
Pakulak 2022	0.68	2.35	7	1.48	3.43	6	5.0%	-0.26 [-1.35, 0.84]	2022
**Subtotal (95% CI)**			**7**			**6**	**5.0%**	**-0.26 [-1.35, 0.84]**	
Heterogeneity: Not applicable									
Test for overall effect: Z = 0.46 (P = 0.65)									
**Total (95% CI)**			**130**			**133**	**100.0%**	**-0.16 [-0.41, 0.08]**	
Heterogeneity: Tau² = 0.00; Chi² = 6.10, df = 11 (P = 0.87); I² = 0%									
Test for overall effect: Z = 1.31 (P = 0.19)									
Test for subgroup differences: Chi² = 0.45, df = 2 (P = 0.80), I² = 0%									

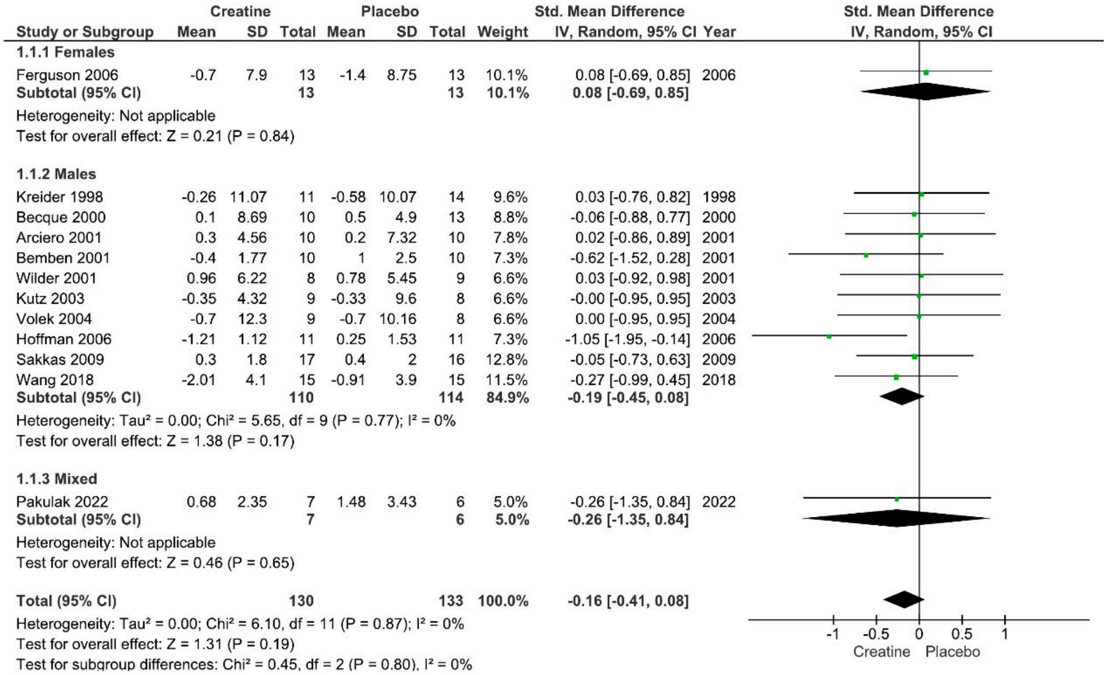

Figure S4. Subgroup analysis based on sex (females vs. males vs. mixed).

Study or Subgroup	Creatine Mean	Creatine SD	Creatine Total	Placebo Mean	Placebo SD	Placebo Total	Weight	Std. Mean Difference IV, Random, 95% CI	Year
**1.1.1 5 g/day and above**									
Kreider 1998	-0.26	11.07	11	-0.58	10.07	14	9.6%	0.03 [-0.76, 0.82]	1998
Arciero 2001	0.3	4.56	10	0.2	7.32	10	7.8%	0.02 [-0.86, 0.89]	2001
Kutz 2003	-0.35	4.32	9	-0.33	9.6	8	6.6%	-0.00 [-0.95, 0.95]	2003
Hoffman 2006	-1.21	1.12	11	0.25	1.53	11	7.3%	-1.05 [-1.95, -0.14]	2006
Pakulak 2022	0.68	2.35	7	1.48	3.43	6	5.0%	-0.26 [-1.35, 0.84]	2022
**Subtotal (95% CI)**			**48**			**49**	**36.2%**	**-0.24 [-0.65, 0.17]**	
Heterogeneity: Tau² = 0.00; Chi² = 4.09, df = 4 (P = 0.39); I² = 2%									
Test for overall effect: Z = 1.13 (P = 0.26)									
**1.1.2 Less than 5 g/day**									
Becque 2000	0.1	8.69	10	0.5	4.9	13	8.8%	-0.06 [-0.88, 0.77]	2000
Bemben 2001	-0.4	1.77	10	1	2.5	10	7.3%	-0.62 [-1.52, 0.28]	2001
Wilder 2001	0.96	6.22	8	0.78	5.45	9	6.6%	0.03 [-0.92, 0.98]	2001
Volek 2004	-0.7	12.3	9	-0.7	10.16	8	6.6%	0.00 [-0.95, 0.95]	2004
Ferguson 2006	-0.7	7.9	13	-1.4	8.75	13	10.1%	0.08 [-0.69, 0.85]	2006
Sakkas 2009	0.3	1.8	17	0.4	2	16	12.8%	-0.05 [-0.73, 0.63]	2009
Wang 2018	-2.01	4.1	15	-0.91	3.9	15	11.5%	-0.27 [-0.99, 0.45]	2018
**Subtotal (95% CI)**			**82**			**84**	**63.8%**	**-0.12 [-0.43, 0.18]**	
Heterogeneity: Tau² = 0.00; Chi² = 1.82, df = 6 (P = 0.94); I² = 0%									
Test for overall effect: Z = 0.78 (P = 0.43)									
**Total (95% CI)**			**130**			**133**	**100.0%**	**-0.16 [-0.41, 0.08]**	
Heterogeneity: Tau² = 0.00; Chi² = 6.10, df = 11 (P = 0.87); I² = 0%									
Test for overall effect: Z = 1.31 (P = 0.19)									
Test for subgroup differences: Chi² = 0.19, df = 1 (P = 0.66), I² = 0%									

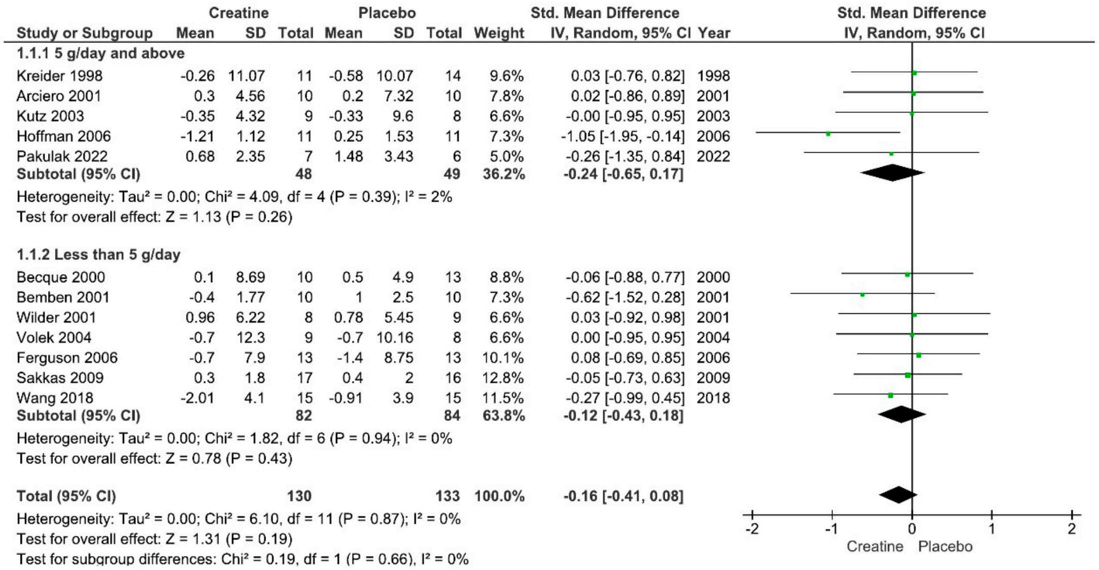

Figure S5. Subgroup analysis based on creatine dose (<5 g vs. ≥5 g).

Study or Subgroup	Creatine Mean	Creatine SD	Creatine Total	Placebo Mean	Placebo SD	Placebo Total	Weight	Std. Mean Difference IV, Random, 95% CI	Year
**1.1.1 8 weeks and above**									
Wilder 2001	0.96	6.22	8	0.78	5.45	9	6.6%	0.03 [-0.92, 0.98]	2001
Bemben 2001	-0.4	1.77	10	1	2.5	10	7.3%	-0.62 [-1.52, 0.28]	2001
Ferguson 2006	-0.7	7.9	13	-1.4	8.75	13	10.1%	0.08 [-0.69, 0.85]	2006
Hoffman 2006	-1.21	1.12	11	0.25	1.53	11	7.3%	-1.05 [-1.95, -0.14]	2006
Sakkas 2009	0.3	1.8	17	0.4	2	16	12.8%	-0.05 [-0.73, 0.63]	2009
**Subtotal (95% CI)**			**59**			**59**	**44.2%**	**-0.28 [-0.69, 0.13]**	
Heterogeneity: Tau² = 0.04; Chi² = 5.00, df = 4 (P = 0.29); I² = 20%									
Test for overall effect: Z = 1.33 (P = 0.18)									
**1.1.2 Below 8 weeks**									
Kreider 1998	-0.26	11.07	11	-0.58	10.07	14	9.6%	0.03 [-0.76, 0.82]	1998
Becque 2000	0.1	8.69	10	0.5	4.9	13	8.8%	-0.06 [-0.88, 0.77]	2000
Arciero 2001	0.3	4.56	10	0.2	7.32	10	7.8%	0.02 [-0.86, 0.89]	2001
Kutz 2003	-0.35	4.32	9	-0.33	9.6	8	6.6%	-0.00 [-0.95, 0.95]	2003
Volek 2004	-0.7	12.3	9	-0.7	10.16	8	6.6%	0.00 [-0.95, 0.95]	2004
Wang 2018	-2.01	4.1	15	-0.91	3.9	15	11.5%	-0.27 [-0.99, 0.45]	2018
Pakulak 2022	0.68	2.35	7	1.48	3.43	6	5.0%	-0.26 [-1.35, 0.84]	2022
**Subtotal (95% CI)**			**71**			**74**	**55.8%**	**-0.08 [-0.41, 0.25]**	
Heterogeneity: Tau² = 0.00; Chi² = 0.54, df = 6 (P = 1.00); I² = 0%									
Test for overall effect: Z = 0.48 (P = 0.63)									
**Total (95% CI)**			**130**			**133**	**100.0%**	**-0.16 [-0.41, 0.08]**	
Heterogeneity: Tau² = 0.00; Chi² = 6.10, df = 11 (P = 0.87); I² = 0%									
Test for overall effect: Z = 1.31 (P = 0.19)									
Test for subgroup differences: Chi² = 0.55, df = 1 (P = 0.46), I² = 0%									

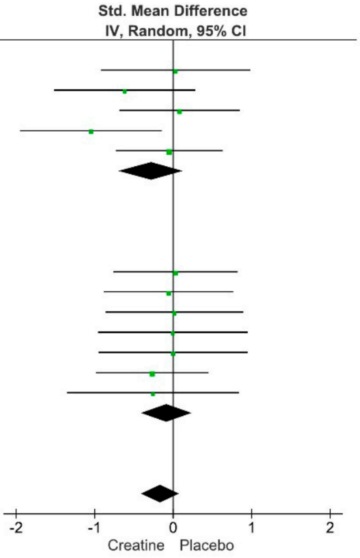

Figure S6. Subgroup analysis based on duration of supplementation (<8 weeks vs. ≥8 weeks).

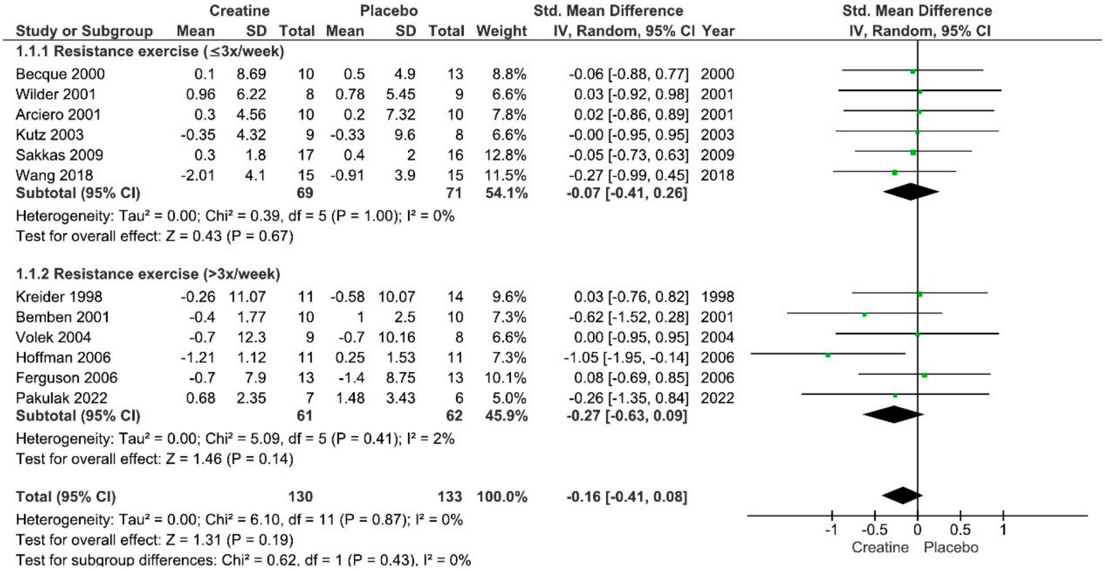

Figure S7. Subgroup analysis based on resistance exercise frequency (≤3x/wk vs. >3x/wk).

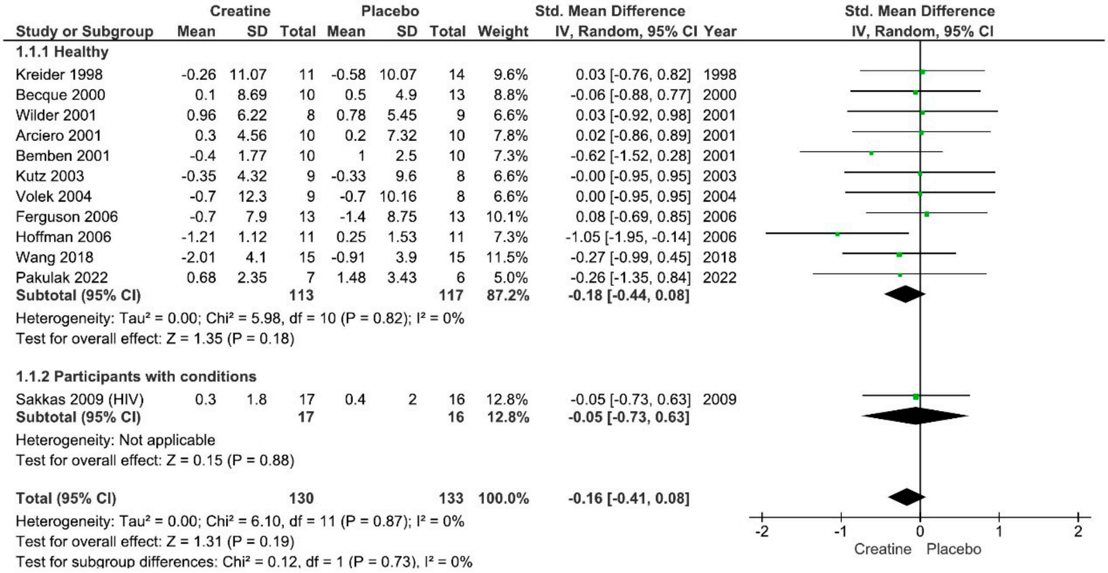

Figure S8. Sensitivity analysis excluding participants with health conditions.

Study or Subgroup	Creatine Mean	Creatine SD	Creatine Total	Placebo Mean	Placebo SD	Placebo Total	Weight	Std. Mean Difference IV, Random, 95% CI	Year
**1.1.1 Assessed dietary intake**									
Kreider 1998	-0.26	11.07	11	-0.58	10.07	14	9.6%	0.03 [-0.76, 0.82]	1998
Arciero 2001	0.3	4.56	10	0.2	7.32	10	7.8%	0.02 [-0.86, 0.89]	2001
Bemben 2001	-0.4	1.77	10	1	2.5	10	7.3%	-0.62 [-1.52, 0.28]	2001
Kutz 2003	-0.35	4.32	9	-0.33	9.6	8	6.6%	-0.00 [-0.95, 0.95]	2003
Volek 2004	-0.7	12.3	9	-0.7	10.16	8	6.6%	0.00 [-0.95, 0.95]	2004
Ferguson 2006	-0.7	7.9	13	-1.4	8.75	13	10.1%	0.08 [-0.69, 0.85]	2006
Hoffman 2006	-1.21	1.12	11	0.25	1.53	11	7.3%	-1.05 [-1.95, -0.14]	2006
Pakulak 2022	0.68	2.35	7	1.48	3.43	6	5.0%	-0.26 [-1.35, 0.84]	2022
**Subtotal (95% CI)**			**80**			**80**	**60.3%**	**-0.20 [-0.52, 0.11]**	
Heterogeneity: Tau² = 0.00; Chi² = 5.63, df = 7 (P = 0.58); I² = 0%									
Test for overall effect: Z = 1.27 (P = 0.20)									
**1.1.2 Did not assess dietary intake**									
Becque 2000	0.1	8.69	10	0.5	4.9	13	8.8%	-0.06 [-0.88, 0.77]	2000
Wilder 2001	0.96	6.22	8	0.78	5.45	9	6.6%	0.03 [-0.92, 0.98]	2001
Sakkas 2009	0.3	1.8	17	0.4	2	16	12.8%	-0.05 [-0.73, 0.63]	2009
Wang 2018	-2.01	4.1	15	-0.91	3.9	15	11.5%	-0.27 [-0.99, 0.45]	2018
**Subtotal (95% CI)**			**50**			**53**	**39.7%**	**-0.10 [-0.49, 0.29]**	
Heterogeneity: Tau² = 0.00; Chi² = 0.31, df = 3 (P = 0.96); I² = 0%									
Test for overall effect: Z = 0.52 (P = 0.61)									
**Total (95% CI)**			**130**			**133**	**100.0%**	**-0.16 [-0.41, 0.08]**	
Heterogeneity: Tau² = 0.00; Chi² = 6.10, df = 11 (P = 0.87); I² = 0%									
Test for overall effect: Z = 1.31 (P = 0.19)									
Test for subgroup differences: Chi² = 0.16, df = 1 (P = 0.69), I² = 0%									

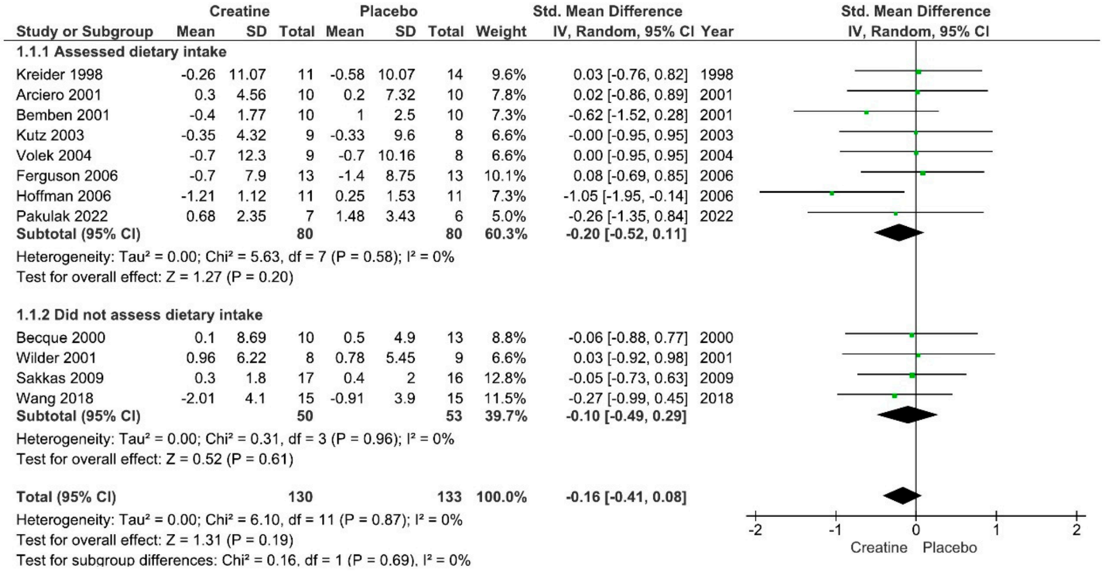

Figure S9. Sensitivity analysis excluding studies that did not assess dietary intake.

Study or Subgroup	Creatine Mean	Creatine SD	Creatine Total	Placebo Mean	Placebo SD	Placebo Total	Weight	Std. Mean Difference IV, Random, 95% CI	Year
Kreider 1998	-0.26	11.07	11	-0.58	10.07	14	11.3%	0.03 [-0.76, 0.82]	1998
Becque 2000	0.1	8.69	10	0.5	4.9	13		Not estimable	2000
Wilder 2001	0.96	6.22	8	0.78	5.45	9	7.8%	0.03 [-0.92, 0.98]	2001
Arciero 2001	0.3	4.56	10	0.2	7.32	10	9.2%	0.02 [-0.86, 0.89]	2001
Bemben 2001	-0.4	1.77	10	1	2.5	10	8.7%	-0.62 [-1.52, 0.28]	2001
Kutz 2003	-0.35	4.32	9	-0.33	9.6	8		Not estimable	2003
Volek 2004	-0.7	12.3	9	-0.7	10.16	8	7.8%	0.00 [-0.95, 0.95]	2004
Ferguson 2006	-0.7	7.9	13	-1.4	8.75	13	11.9%	0.08 [-0.69, 0.85]	2006
Hoffman 2006	-1.21	1.12	11	0.25	1.53	11	8.7%	-1.05 [-1.95, -0.14]	2006
Sakkas 2009	0.3	1.8	17	0.4	2	16	15.1%	-0.05 [-0.73, 0.63]	2009
Wang 2018	-2.01	4.1	15	-0.91	3.9	15	13.6%	-0.27 [-0.99, 0.45]	2018
Pakulak 2022	0.68	2.35	7	1.48	3.43	6	5.9%	-0.26 [-1.35, 0.84]	2022
**Total (95% CI)**			**111**			**112**	**100.0%**	**-0.19 [-0.45, 0.08]**	
Heterogeneity: Tau² = 0.00; Chi² = 5.89, df = 9 (P = 0.75); I² = 0%									
Test for overall effect: Z = 1.38 (P = 0.17)									

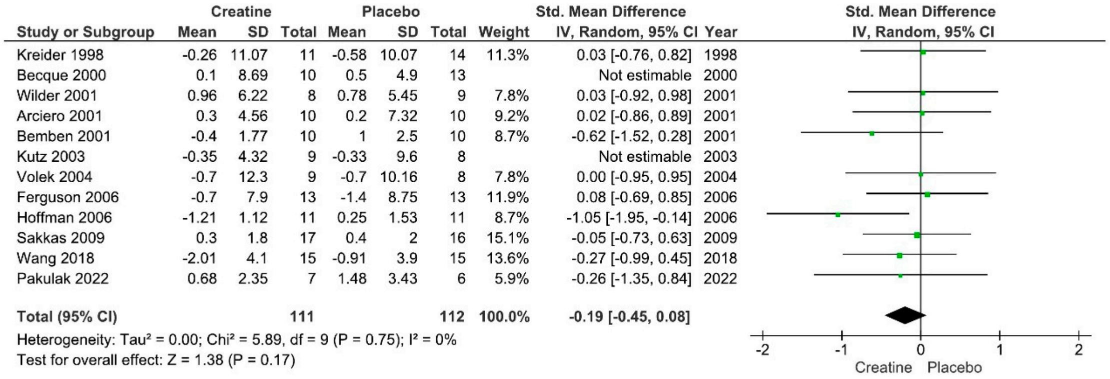

Figure S10. Sensitivity analysis excluding studies with increased risk of bias.
